# Cold Physical Plasma Decreases the Viability of Lung Adenocarcinoma Cells

**DOI:** 10.32607/20758251-2019-11-3-16-19

**Published:** 2019

**Authors:** E. A. Golubitskaya, O. S. Troitskaya, E. V. Yelak, P. P. Gugin, V. A. Richter, I. V. Schweigert, D. E. Zakrevsky, O. A. Koval

**Affiliations:** Institute of Chemical Biology and Fundamental Medicine, Siberian Branch of the Russian Academy of Sciences, Akad. Lavrentiev Ave. 8, Novosibirsk, 630090, Russia; Novosibirsk State University, Pirogova Str. 1, Novosibirsk, 630090, Russia; Novosibirsk State Technical University, K. Marx Ave. 20, Novosibirsk, 630073, Russia; Rzhanov Institute of Semiconductor Physic, Siberian Branch of the Russian Academy of Sciences, Akad. Lavrentiev Ave. 13, Novosibirsk, 630090, Russia; Khristianovich Institute of Theoretical and Applied Mechanics, Siberian Branch of the Russian Academy of Sciences, Institutskaya Str. 4/1, Novosibirsk, 630090, Russia

**Keywords:** cold atmospheric plasma, antitumor therapy, reactive oxygen species, lung adenocarcinoma

## Abstract

The high mortality rate that accompanies cancer spurs the search for new methods that can be used to treat malignant neoplasms. In addition to chemotherapy, electrophysical techniques for tumor treatment appear rather promising. The results of in vitro exposure of A549 human lung adenocarcinoma cells to cold atmospheric plasma (CAP) are hereby presented. A gas-discharge device that generates a sequence of streamers propagating along a stream of inert gas in the ambient air was used. In the zone where the plasma jet came into contact with the target object, there were high-intensity electric fields and high plasma concentrations, while the gas temperature changed by less than a degree. In this study, we compared the cytotoxic effect of CAP in helium and argon. Direct irradiation of cells by CAP with U = 4.2 kV for 30–120 s was shown to reduce cell viability by 25%. Variation of the amplitude of the AC voltage in the plasma device in argon within a range of 3.8–5.6 kV did not significantly alter the cell death rate. Further optimization of the modes of CAP generation in gas-discharge devices with various geometries for the trea

## INTRODUCTION

Along with the development of efficacious chemotherapeutic agents to treat malignant neoplasms, much attention is currently being focused on physical methods such as radiotherapy (including photon radiation therapy, proton beam therapy, and boron neutron capture therapy) [1, 2]. The application of cold atmospheric plasma (CAP) is among the promising novel biophysical approaches to the treatment of a number of malignancies [3]. The potential risk factors associated with the use of CAP on humans include the risk that some amount of current may flow through tissue, thermal damage, and exposure to UV radiation. However, all these drawbacks can be eliminated at the stage of selection of gas composition, radiation intensity, and duration. CAP has been shown to be safe in patients with chronic skin ulcers [4, 5]. CAP is a sequence of streamers that are generated in inert gases in the dielectric channel of a plasma jet device and propagate along the gas stream in ambient air under atmospheric pressure. A gas mixture consisting of an inert gas, nitrogen, oxygen, and water vapor is excited and ionized thanks to high-energy electrons and the high plasma concentration in the steamer head, where the electric field intensity can go as high as 10–20 kV/cm. Various oxygen- and nitrogen-containing compounds, such as H_2_O_2_, HNO_2_, HNO_3_, N_2_O, NO_2_, NO, and N_2_O_3_, form in plasma-stimulated chemical reactions. Inert gases are commonly used as a working gas in plasma jet devices, since air breakdown voltage is much higher. The low temperature in the zone where cold plasma comes into contact with a biological object is an attractive feature of the technique of using a plasma jet in antitumor approaches [3]. Cold atmospheric plasma was shown to exhibit cytotoxic activity against more than 20 lines of tumor cells of different histogenesis and in experimental in vivo models in animals carrying tumors [6]. Reactive oxygen (ROS) and reactive nitrogen species (RNS) were identified as the key molecules that trigger cell death upon exposure to CAP. H_2_O_2_ molecules are believed to play a crucial role among ROS as they can induce mitochondrial and DNA damage [7]. It was shown experimentally that cells undergo a synergistic effect of H_2_O_2_, NO_2_ - and NO_3_ - upon exposure to CAP, but the cytotoxicity of RNS is much lower than that of ROS [8]. Not only does CAP treatment induce cytotoxic effects, but it also may restore the susceptibility of resistant tumor cells to cytostatics (e.g., in case of temozolomide-resistant glioblastoma) [9].

**Fig. 1:**
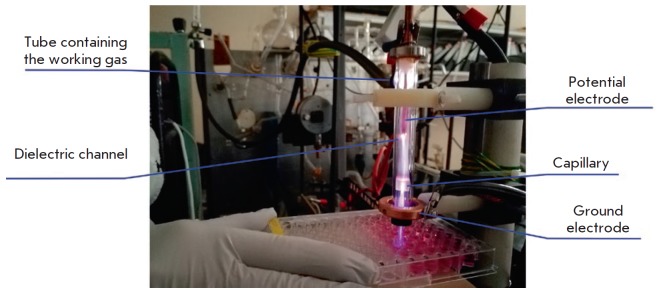
The plasma jet device for the irradiation of cultured cells

In this study, we used an original device that generates a plasma jet, which allows one to widely vary the modes of streamer breakdown in the dielectric channel and the dynamics of streamer sequence propagation along an inert gas jet (Fig. 1). The effects of the exposure of human tumor cells to CAP in helium and argon were compared. The reason for using two working gases was that argon and helium have different physical properties (namely, argon atoms are tenfold heavier than helium atoms, while the threshold ionization and excitation energies are lower for argon), which eventually influences the physical properties of the plasma jet (electron concentration, electric field intensity, etc.) and the near-surface plasma chemistry.

## EXPERIMENTAL

Our gas-discharge device consisted of a dielectric coaxial channel (100 mm long) with an inside diameter of 8 mm. A metal (copper) electrode (50mm long and 2 mm in diameter) and a capillary (6 mm long; inside diameter, 2.6 mm) were coaxially inserted into the channel and immobilized with a quartz bushing (23 mm long; inside diameter, 5 mm). The quartz channel was surrounded by an annular copper electrode. The discharge zone consisted of an inner (recording) and outer (ground) electrode. A sinusoidal voltage (frequency, ~ 25 kHz and amplitude < 6 kV) produced by a high-voltage pulse generator was applied to the recording electrode. The gas system ensured a flow rate of working gases of up to 15 l/min at an excessive pressure in the gas line of 1 atm. The experiments were conducted using helium and argon as working gases.

As the working gas is fed and the sinusoidal voltage U applied, a breakdown is observed during the positive half-wave between the recording and ground electrodes. As the voltage U is further increased to > 1–3 kV (depending on the gas and gas flow rate), a plasma jet is formed. This jet leaves the dielectric channel and propagates in free space. Depending on the excitation parameters, a typical jet length is 5–50 mm (in helium) and 5–20 mm (in argon).

A549 human lung adenocarcinoma cells (Russian Collection of Cell Cultures Vertebrate, Institute of Cytology, St. Petersburg, Russia) were used in this study. The cells were cultured in DMEM (GIBCO, USA) supplemented with 2 mM L-glutamine (Sigma-Aldrich, USA), 10% FBS (GIBCO, USA), and an antibioticantimycotic solution (100 U/ml penicillin, 100 mg/ml streptomycin sulfate, 0.25 μg/ml amphotericin; GIBCO, USA) at 37.0 ± 1.0°C under an atmosphere of 5.0 ± 0.5% CO_2_.

The cells to be further exposed to CAP were cultured in 96-well plates (4 × 10^3^ cells per well) in 100 μL. Once the cell monolayer had reached 70–80% confluence, the cells were exposed to cold atmospheric plasma treatment. The cells were cultured under standard conditions for 24 days. The medium was then replaced with a serum-free RPMI medium supplemented with 0.25 mg/ml MTT (3-(4,5-dimethylthiazol-2-yl)-2,5- diphenyltetrazolium bromide) (Sigma, USA), and the cells were incubated for 4 h at 37°C. Next, the medium was removed from the wells and formazan crystals were dissolved in DMSO. The optical density of the solution in the wells was measured on a multichannel spectrophotometer (Berthold Technologies, Germany) at λ = 570 nm.

## RESULTS AND DISCUSSION

**Fig. 2:**
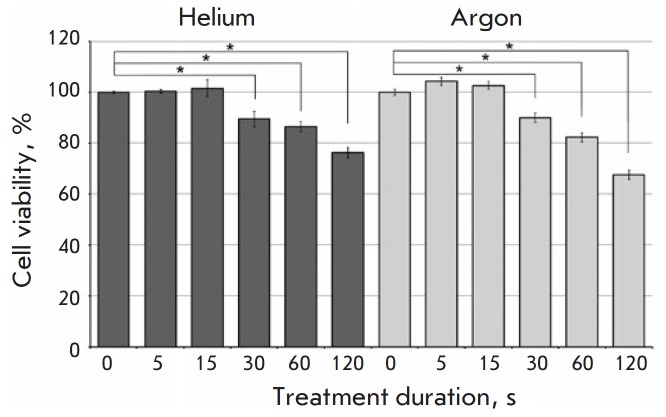
The effect of cold physical plasma on the viability of A549 adenocarcinoma cells. Voltage, 4.2 kV. The viability of the control (untreated) cells was 100%. The MTT assay data are presented as a mean of three independent experiments ± SD, *p < 0.05

The effect of a cold atmospheric plasma on the survival of tumor cells was studied using the A549 human lung adenocarcinoma cell line. It was demonstrated by MTT assay that cell viability decreases as treatment duration is increased from 5 s to 2 min. Short-term ( < 30 s) CAP treatment of the cells had virtually no effect on cell survival (Fig. 2). The maximum reduction of cell viability was attained after a 2-min treatment phase, both for helium and for argon.

Cell death was visualized using propidium iodide (PI), a low-molecular-weight fluorescent dye that is capable of intercalating into the DNA of dying cells with the damaged membrane but does not penetrate into living cells. It was found that the percentage of stained cells increases with CAP treatment duration (the data are not shown). Hence, it was demonstrated that CAP treatment induces cell death.

**Fig. 3:**
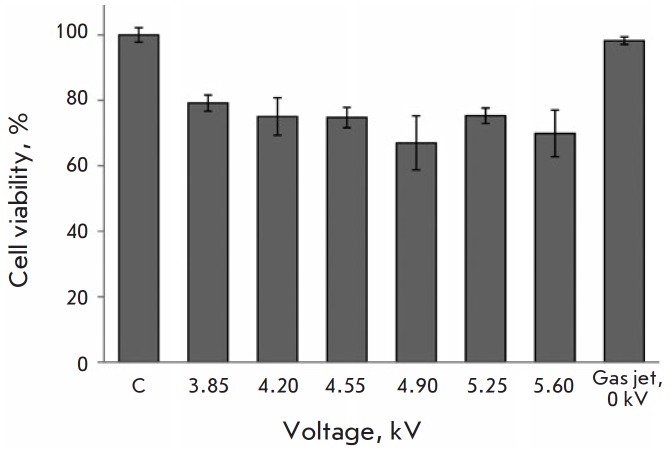
Voltage-dependent cytotoxicity of CAP irradiation of A549 cells in argon. The MTT assay data 48h post-irradiation. C – control (untreated) cells. The data are presented as a mean of three independent experiments ± SD

When using a plasma jet, the loaded electrode voltage is one of the key parameters responsible for plasma jet dynamics and the plasma–surface interaction. The energy of the electrons in the plasma jet, as well as the rates of ionization, dissociation, and excitation of molecules due to electron impact, depends on the applied pressure, which may have a significant effect on the induced plasma-chemical processes and, therefore, the biological effect of a plasma jet. Direct irradiation at various AC voltage amplitudes was employed to analyze the effect of the voltage applied to the loaded electrode in a plasma jet device generating CAP on cytotoxic activity against A549 cells. It was found experimentally that changes in the voltage amplitude of argon within a range of 3.8–5.6 kV have no effect on the level of cell death (Fig. 3). Additional studies focused on the impact of an inert gas flow without a plasma jet on cells and studies at zero voltage demonstrated that both the argon and helium flows did not affect the viability of the irradiated cells.

## CONCLUSIONS

Hence, it has been demonstrated for A549 lung adenocarcinoma cells that direct exposure to cold atmospheric plasma causes tumor cell death. Variation of the modes of the irradiating inert gases (including their mixtures with molecular gases) in the gas-discharge device allows one to generate a cold plasma jet with a wide range of energy parameters and compositions, which certainly is an advantage of this study. Further optimization of devices based on dielectric channels of different geometries, as well as the mediated treatment of cells through the pre-irradiated culture medium, will make it possible to choose the optimal conditions for the impact of cold atmospheric plasma on human tumor cells. The safety of CAP treatment as relates to human tissue has already been demonstrated; therefore, it is beyond any doubt that this method can potentially be used in clinical practice to treat certain malignant neoplasms.

## Abbreviations

CAP: cold atmospheric plasma
FBS: fetal bovine serum
MTT: 3-(4,5-dimethylthiazol-2-yl)-2,5-diphenyltetrazolium bromide
RNS: reactive nitrogen species
ROS: reactive oxygen species
